# Influence of Selected Ophthalmic Fluids on the Wettability and Hydration of Hydrogel and Silicone Hydrogel Contact Lenses—In Vitro Study

**DOI:** 10.3390/ma15030930

**Published:** 2022-01-25

**Authors:** Gabriela Chwalik-Pilszyk, Anna Wiśniewska

**Affiliations:** Department of Applied Mechanics and Biomechanics, Faculty of Mechanical Engineering, Tadeusz Kosciuszko Cracow University of Technology, 37 Jana Pawla II Av., 31-864 Cracow, Poland

**Keywords:** surface free energy (SFE), contact angle (CA), contact lenses, wettability, dehydration

## Abstract

This study attempts to evaluate the effect of incubation in selected ophthalmic fluids on contact lenses (Etafilcon A, Omafilcon A, Narafilcon A, Senofilcon A). Four research groups differing in the incubation environment were created: (1) initial state, (2) contact lens solution (CLS), (3) contact lens solution and eye drops (ED) and (4) eye drops. Dehydration by gravimetric method and the contact angle (CA) by the sessile drop method were tested. The surface free energy (SFE) was also calculated with the use of several methods: Owens–Wendt, Wu, Neumann, and Neumann–Kwok. The greatest changes in the dehydration profile were observed for contact lenses incubated in ED. The most noticeable changes in CA values were observed for contact lenses incubated in ED, in which it was not possible to settle water drop after incubation. On the basis of SFE analysis, higher values were found for hydrogel contact lenses, e.g., according to the Owens–Wendt method, they ranged from 54.45 ± 6.56 mJ/m${}^{2}$ to 58.09 ± 4.86 mJ/m${}^{2}$, while in the case of silicone-hydrogel contact lenses, they ranged from 32.86 ± 3.47 mJ/m${}^{2}$ to 35.33 ± 6.56 mJ/m${}^{2}$. Incubation in all tested environments decreased the SFE values, but the differences were in most cases statistically insignificant. Calculating the SFE may be a useful method as it can be used to estimate the possibility of bacteria adhering to contact lens surfaces.

## 1. Introduction

Many contact lens wearers complain of discomfort caused by the occurrence of dry eyes. Discomfort may also be related to factors such as protein adsorption or friction during blinking, especially between the front side of the lens and the inner eyelid [1,2,3]. Dry eye symptoms are reported by approximately 50% of contact lens wearers, and 25% of patients drop out of contact lenses because of dry eye complications [1,2,4,5,6,7,8,9]. Currently, the main factors influencing the development of materials used in the production of contact lenses are the need to increase the comfort [10].

Some studies show that dry eye symptoms can be better characterized as neuropathic eye pain [11,12]. According to McMonnies [12], some cases of contact lens intolerance may be due to eyelid wiper neuropathy. A stagnant post-lens tear pool with the possible increased concentrations of metabolic by-products, cellular debris, and bacterial exotoxins can disrupt the corneal epithelial and sub-basal nerves. The study of Situ et al. [13] shows that one effect of wearing contact lenses was to alter the functioning of the sensory nerves of the ocular surface, which is reflected in response to stimulation of the cornea and conjunctiva. According to Murphy et al. [14], there is a significant reduction in corneal sensitivity between contact lens wearers and non-lens wearers, with the degree of loss of sensitivity not related to the duration of contact lens wear and appearing to stabilize after the first few months of wearing.

During wearing contact lenses, alternating air drying (open eye) and rehydration (closing the eye when blinking) cause the lens dehydration [15]. The interaction between the contact lens and the cornea significantly affects the biocompatibility of the lens [16]. Dehydration of the contact lens can cause discomfort because the water molecules attached to the lens surface reduce friction, preventing eye irritation [10]. Additionally, it can alter some important properties such as radius of curvature, thickness, oxygen permeability [17], and surface wettability [2,9,18,19]. Many factors affect the rate of dehydration, including the environment [20,21,22,23], water content [21,24], water binding properties [25,26], thickness [27], surfactant use [28], and contact lens time of wear [29].

According to Jones et al. [8], environmental conditions have a significant influence on the dehydration of the contact lenses. In vitro studies have shown that higher airflow has a greater influence on the dehydration rate (DR) than its humidity [8]. Note that in vitro dehydration differs significantly from in vivo dehydration via other mechanisms [29] and due to external factors such as temperature, osmolarity, pH, etc. [30].

The DR varies depending on the contact lens material and affects wearer comfort [28,31]. The interaction between the polymer chains and water influences the physical properties of the hydrated lens system [32]. The hydrated state of a hydrogel contact lens is created by the interaction of polymer chains with water molecules. The structure and physical properties of these materials are thus a result of the existence of water binding sites in the polymer and the geometric constraints [33]. González-Méijome et al. [30] observed that HEMA-based contact lenses dehydrate to a greater extent and faster than silicone hydrogel materials. This is consistent with the research of Martín-Montañez et al. [34], in which it was found that silicone hydrogel contact lenses (Balafilcon A, Lotrafilcon B, Senofilcon A) have a longer stabilization time, which indicates that these materials might have higher ability to avoid dehydration.

Krysztofiak [32] distinguishes three phases of contact lens dehydration, differing in terms of the DR: The dehydration rate in phase I is the most intense; phase II usually lasts the same time regardless of the type of contact lens. The duration of phase III depends on whether in vitro or in vivo tests are being conducted [32].

To reduce dryness and irritation, eye drops (ED) are recommended. It is the first-line treatment for dry eye symptoms [9,35]. Approximately 20% of contact lens wearers use ED to relieve discomfort [36]. Most eye drops contain sodium hyaluronate as the main lubricant and soothing agent [6,9]. Hyaluronic acid (HA) is a linear biopolysaccharide with exceptional hygroscopic, lubricating, and viscoelastic properties [6,37]. In the eye, HA protects the corneal epithelium, accelerates cell migration and wound healing [38], and also has a unique ability to attract and retain water [37,39]. Studies have shown that HA-containing drops have a positive effect on the treatment of dry eye symptoms [39,40,41]. However, contact lens wear discomfort and dryness are still unsolved issues for a large number of wearers as these wetting agents only have a temporary effect on lubricity [7].

**Figure 1:**
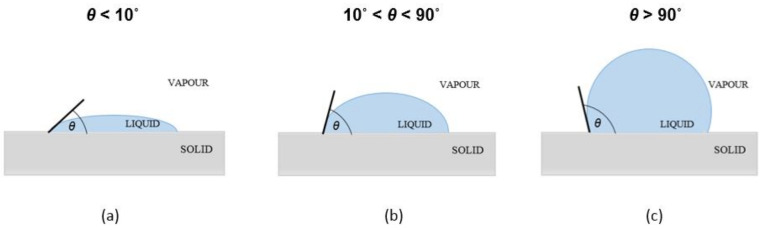
Different cases of liquid contact angle on surfaces specifying wettability: (**a**) high wettability, (**b**) medium wettability, and (**c**) low wettability.

Surface wettability is essential for the physiological compatibility between the contact lens and the eye as normal corneal hydration is disrupted by the insertion of the contact lens [28]. Wettability can be assessed by contact angle (CA) measurements (Figure 1). Analysis of CA can be used to calculate the surface free energy (SFE), affording information about the character of a surface [42,43]. The surface is considered hydrophilic when the value of water contact angle is less than 90${}^{{}^{\circ}}$ [44].

The overall trend suggests that a decrease in CA correlates with a decrease in the friction coefficient [7]. Obtaining the hydrophilic surface in the contact lens is essential in terms of comfort and security, which is related to lens dehydration and the accumulation of the deposits [45,46]. Results obtained by Giraldez et al. [47] suggest that more hydrophobic contact lenses are more prone to *S. epidermidis* adhesion. According to Yu et al. [7], the use of hydrophilic polymers enhances the wearing comfort of the contact lens due to better wettability. This modification can improve lubricity [48]. Low surface tension reduces protein adsorption from body fluids, which prevents inflammation [32].

This study aimed to evaluate the effect of contact lens solution (CLS) and hyaluronan eye drops on the dehydration process and the surface properties of hydrogel and silicone hydrogel contact lenses available on the market. The gravimetric method was used to assess the dehydration process. The equilibrium water content (EWC) and dehydration rate were calculated. Based on the contact angle measurements, the surface free energy was calculated which allowed to determine the character of the material’s surface.

## 2. Materials and Methods

### 2.1. Contact Lenses

Four commercial contact lenses materials were tested: hydrogel (Etafilcon A, Omafilcon A) and silicone hydrogel (Narafilcon A, Senofilcon A) which were characterized in Table 1. To avoid the influence of the differences in contact lens thickness on the dehydration result, all the lenses used were −2.50 D back vertex power.

### 2.2. Sample Preparation

For all tests, the contact lenses were prepared using the same protocol to ensure consistent starting conditions. The contact lens was removed from the storage solution with tweezers, touching only the edges of the lens and taking care not to deposit any contamination on the material. The sample was then rinsed in saline and tested immediately or placed in the solution in which the contact lenses were incubated.

Four study groups, each with 10 contact lenses, were formed. The first set was a reference group consisting of contact lenses taken directly from the package. The second group included samples incubated in the CLS with the following composition: 0.03% hydroxyalkylphosphonate, boric acid, sodium edetate, 1% poloxamine, sodium borate, sodium chloride, and 0.0001% polyaminopropyl biguanide. The third group consisted of samples incubated in the CLS, to which 50 µL ED containing trehalose (3 mg/mL) and hyaluronan (1.5 mg/mL) were added every 1 h. The fourth samples set was incubated in ED. Incubation was conducted at 37 ${}^{{}^{\circ}}$C for 8h in the Advantage-Lab E2 oven (Advantage-Lab, Schilde, Belgium).

### 2.3. Dehydration

The gravimetric method was used to perform a qualitative and quantitative description of the dehydration process. The contact lenses were removed from the incubation solution (the reference group was taken directly from the package) and then put on a convex plastic holder. Its curvature was close to the radius of the contact lens to recreate the conditions on the eye (only the front surface exposed to air). Gravimetric measurement was started after placing the sample on an analytical balance (RADWAG AS 160/C/2, RADWAG, Radom, Poland) with an accuracy of $10^{-4}$ g and the contact lens mass was recorded every minute. Measurements were made at 23 ${}^{{}^{\circ}}$C and 55% relative humidity. The moment when the mass of the sample did not change for at least 5 min was assumed as the equilibrium state. The obtained results allowed calculating the equilibrium water content (*EWC*) of the tested samples using Equation (1) [51]:

(1) $$EWC=\frac{\left(m_{0}-m_{f}\right)}{m_{0}}\cdot 100\%$$

where $m_{0}$ is the initial sample mass and $m_{f}$ is the sample mass after complete dehydration.

Then dehydration rate (*DR*) of the material was determined according to [52]

(2) $$DR=\frac{\left(m_{t}-m_{t-1}\right)}{m_{t}}$$

where $m_{t}$ is the sample mass at time *t* and $m_{t-1}$ is the sample mass at time *t* − 1.

### 2.4. Wettability and Surface Free Energy (SFE)

Based on the CA tests, the wettability was assessed and the values of SFE were determined. The measurements were carried out with a sessile drop method using an optical goniometer (Advex Instrument, Brno-Komin, Czech Republic) connected to a computer equipped with SeeSystem v.6.3 software (Advex Instrument, Brno-Komin, Czech Republic). The contact lenses were placed on a rounded profile with a curvature close to the radius of the corneal curvature and then a 0.4 µL drop of the measuring liquid was applied using a micropipette (VITRUM/VWR, Stribrna Skalice, Czech Republic) with a measuring range of 0.1 ÷ 0.5 µL. Disposable tips with an internal diameter of 0.5 mm were used. To test the CA, diiodomethane (Merck, Warsaw, Poland) and distilled water (Poch, Gliwice, Poland) were used. The values of SFE, as well as polar and dispersive components for these liquids, are presented in Table 2.

**Figure 2:**
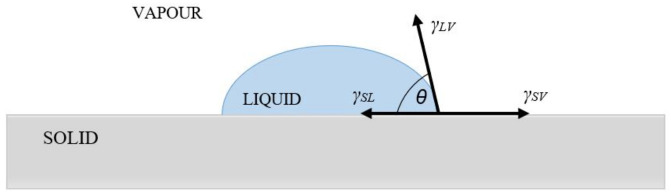
The mechanical equilibrium of the drop on a plane solid surface: $\gamma_{SL}$: SFE between solid and liquid, $\gamma_{LV}$: SFE between liquid and vapor, $\gamma_{SV}$: SFE between solid and vapor.

The angle between a solid and a liquid droplet can be determined from the drop shape using Young’s Equation (3):

(3) $$\gamma_{LV}\cdot cos\theta =\gamma_{SV}-\gamma_{SL}$$

where $\gamma_{LV}$ is the SFE between liquid and vapor, θ is the measured contact angle of liquid, $\gamma_{SV}$ is the SFE between solid and vapor, and $\gamma_{SL}$ is the SFE between solid and liquid (Figure 2).

The optical goniometer captured the drop image and SeeSystem software calculated the value of CA from it based on the width and height of the drop. There were 10 samples in each test group. For every tested contact lens, 10 measurements were made. This gave 100 measurements for each analyzed case, on the basis of which the mean value used for further analysis was calculated.

There are several methods for calculating surface free energy [53]. In this study, selected four theories (Owens–Wendt, Wu, Neumann, and Neumann–Kwok) were used.

#### 2.4.1. Owens–Wendt Method

According to the Owens–Wendt method [54], the SFE is the sum of two components: the dispersive $\gamma_{S}^{d}$ and the polar $\gamma_{S}^{p}$ (4),

(4) $$\gamma_{S}=\gamma_{S}^{d}+\gamma_{S}^{p}$$

where $\gamma_{S}$—SFE of tested material, $\gamma_{S}^{d}$—dispersive component of SFE of tested material, and $\gamma_{S}^{p}$—polar component of SFE of tested material.

To calculate SFE components (dispersive and polar), it is necessary to know the values of the contact angles measured with two liquids. Distilled water is most often used as a polar liquid and diiodomethane as a non-polar liquid [53,55,56]. Calculating the values of both SFE components is possible thanks to the geometric mean method (5):

(5) $$\frac{1}{2}\left(1+cos\theta \right)\gamma_{L}=\sqrt{\gamma_{S}^{d}\gamma_{L}^{d}}+\sqrt{\gamma_{S}^{p}\gamma_{L}^{p}}$$

where θ—measured contact angle, $\gamma_{L}$—SFE of measurement liquid, $\gamma_{S}^{d}$—dispersive component of SFE of tested material, $\gamma_{L}^{d}$—dispersive component of SFE of a liquid, $\gamma_{S}^{p}$—polar component of SFE of tested material, and $\gamma_{L}^{p}$—polar component of SFE of a liquid.

Due to the use of two measuring liquids, a system of two linear equations is obtained: one equation contains the data for a polar liquid and the other one the data for a non-polar liquid.

#### 2.4.2. Wu Method

The Wu approach [57], similarly to the Owens–Wendt method, requires the use of two measuring liquids. Using this method, it is also possible to calculate the value of the SFE and its components (dispersive and polar). The Wu method is also called the harmonic mean method because of the equation used (6):

(6) $$\;\left(1+cos\theta \right)\gamma_{L}=4\left(\frac{\gamma_{L}^{d}\gamma_{S}^{d}}{\gamma_{L}^{d}+\gamma_{S}^{d}}+\frac{\gamma_{L}^{p}\gamma_{S}^{p}}{\gamma_{L}^{p}+\gamma_{S}^{p}}\right)$$

where θ—measured contact angle, $\gamma_{L}$—SFE of measurement liquid, $\gamma_{L}^{d}$—dispersive component of SFE of a liquid, $\gamma_{S}^{d}$—dispersive component of SFE of tested material, $\gamma_{L}^{p}$—polar component of SFE of a liquid, and $\gamma_{S}^{p}$—polar component of SFE of tested material.

The presented equation is most often solved numerically. You can also find an analytical solution, presented, e.g., by Hejda et al. [58].

#### 2.4.3. Neumann Method

Neumann and co-workers have considered the surface tension of solids from a thermodynamic point of view. To determine the value of the SFE, measurements of the contact angle with only one measuring liquid are used [59]. The Neumann method cannot be applied to determine the independent polar and dispersive SFE components. Most often, measurements are conducted using distilled water. Equation (7), that emerged from the modification of the Berthelot hypothesis, is expressed [60] as

(7) $${\left(\frac{\gamma_{S}}{\gamma_{L}}\right)}^{0.5}e^{-\beta_{1}{\left(\gamma_{L}-\gamma_{S}\right)}^{2}}=0.5\left(1+cos\theta \right)$$

where $\gamma_{S}$—SFE of tested material, $\gamma_{L}$—SFE of measurement liquid, coefficient $\beta_{1}=0.0001247$ (determined experimentally), and θ—measured contact angle.

#### 2.4.4. Neumann–Kwok Method

Another modification of the Berthelot hypothesis resulted in the formulation (8) of a new equation by Neumann, this time with Kwok [61]:

(8) $${\left(\frac{\gamma_{S}}{\gamma_{L}}\right)}^{0.5}\left[1-\beta_{2}{\left(\gamma_{L}-\gamma_{S}\right)}^{2}\right]=0.5\left(1+cos\theta \right)$$

where $\gamma_{S}$—SFE of tested material, $\gamma_{L}$—SFE of measurement liquid, coefficient $\beta_{2}=0.0001057$ (determined experimentally), and θ—measured contact angle.

For this variant of the Neumann method, it is also not possible to determine individual components of the SFE, and one measuring liquid is enough to perform the analysis. The result can be calculated using, e.g., Newton’s iterative method [58].

Statistical analysis was conducted using the Student’s *t*-test for two-group cases and the one-way analysis of variance (ANOVA) for differences among at least three-group cases.

## 3. Results

### 3.1. Dehydration

**Figure 3:**
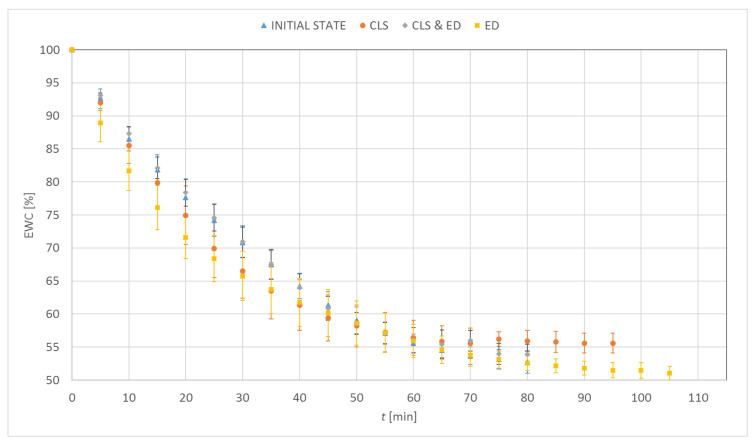
Distribution of equilibrium water content values as a function of time for Etafilcon A.

**Figure 4:**
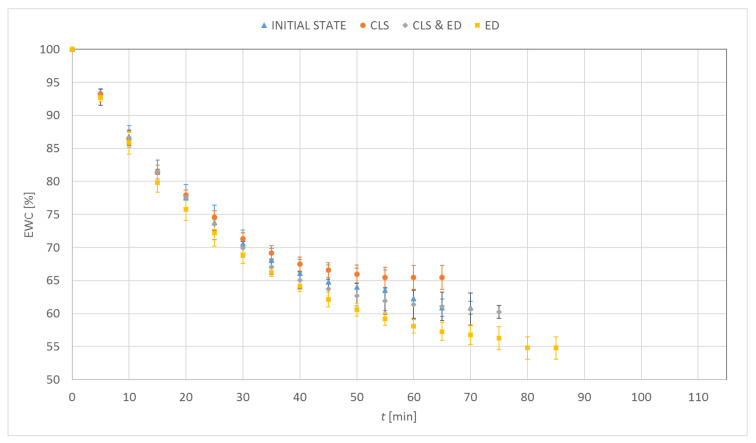
Distribution of equilibrium water content values as a function of time for Omafilcon A.

**Figure 5:**
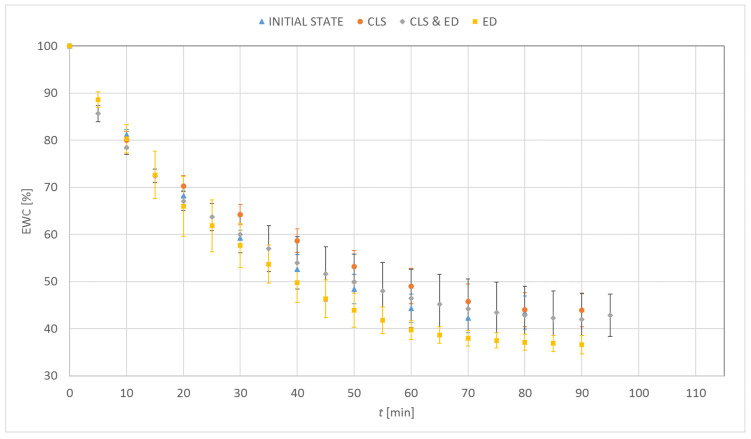
Distribution of equilibrium water content values as a function of time for Narafilcon A.

**Figure 6:**
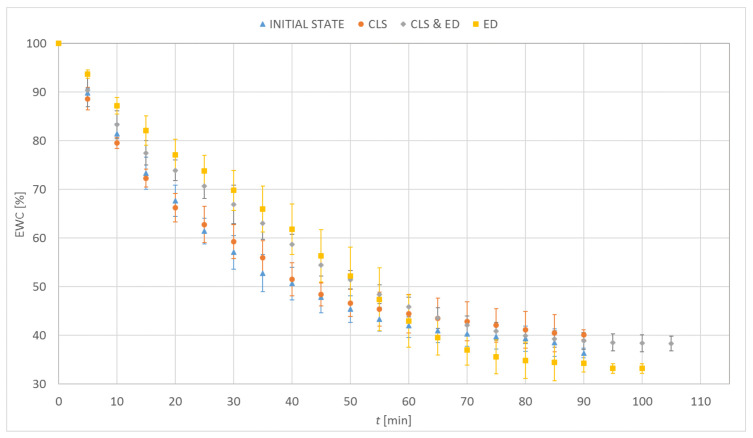
Distribution of equilibrium water content values as a function of time for Senofilcon A.

Figure 3, Figure 4, Figure 5 and Figure 6 show the percentage change in hydration of the tested contact lenses as a function of time. The presented results are the arithmetic mean of all measurements made for a given research group. Three dehydration phases could be observed. The phase I was characterized by the highest DR. In the phase II, a decrease in the DR was observed (stabilization of samples). In the phase III, the hydration of the samples remained constant. Hydrogel contact lenses were characterized by greater hydration. In the initial state, hydration of Etafilcon A was 54.6% and for Omafilcon A was 60.6%, while hydration of Narafilcon A and Senofilcon A was 44.4% and 36.3%, respectively.

The obtained EWC values were similar to the values declared by the manufacturers. The dehydration process was fastest in lenses made of Omafilcon A and lasted 69 min. The longest dehydration process was observed for Senofilcon A (94 min). There was no unequivocal trend regarding the effect of incubation in CLS on dehydration time. However, the addition of HA resulted in the extension of the dehydration time. The addition of ED increased the time needed to dehydrate the contact lenses compared to the reference trial. Additionally, a decrease in the EWC value was observed. In all cases of incubation in CLS, an increase in EWC values was noticed. The difference was significant for Omafilcon A and Senofilcon A (*p* < 0.05).

Less impact of incubation in ED on the dehydration time was observed for silicone hydrogel lenses (for Senofilcon A an increase by 5%, for Narafilcon A by 8%) compared to hydrogel lenses (for Omafilcon A an increase by 20%, for Etafilcon A by 30%). The dehydration time has increased and the EWC values have decreased. In the case of silicone hydrogel contact lenses, the longest dehydration time was observed for samples incubated in CLS & ED, and for hydrogel lenses, it was for the samples stored in ED.

**Figure 7:**
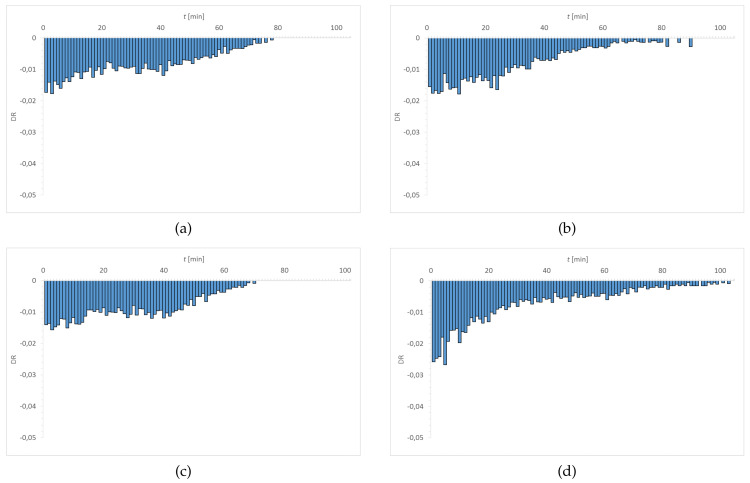
Distribution of DR values as a function of time for Etafilcon A: (**a**) initial state, (**b**) incubated in CLS, (**c**) incubated in CLS & ED, and (**d**) incubated in ED.

**Figure 8:**
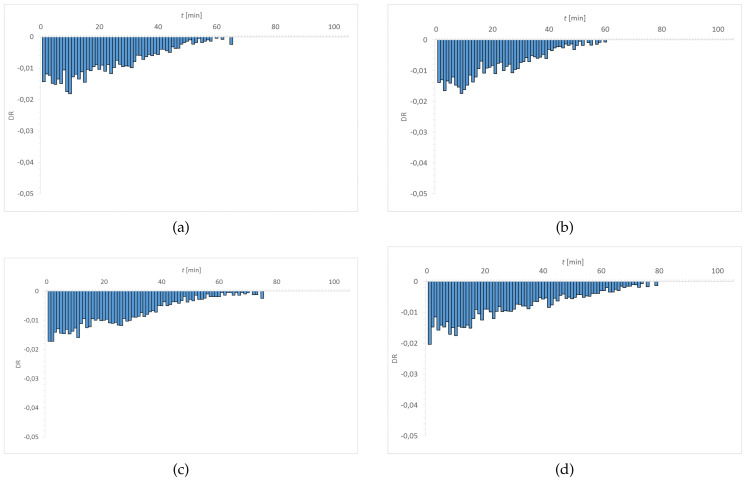
Distribution of DR values as a function of time for Omafilcon A: (**a**) initial state, (**b**) incubated in CLS, (**c**) incubated in CLS & ED, and (**d**) incubated in ED.

**Figure 9:**
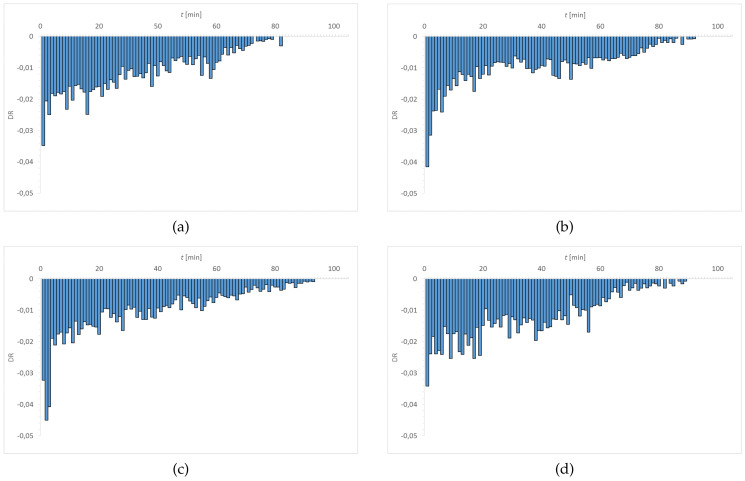
Distribution of DR values as a function of time for Narafilcon A: (**a**) initial state, (**b**) incubated in CLS, (**c**) incubated in CLS & ED, and (**d**) incubated in ED.

**Figure 10:**
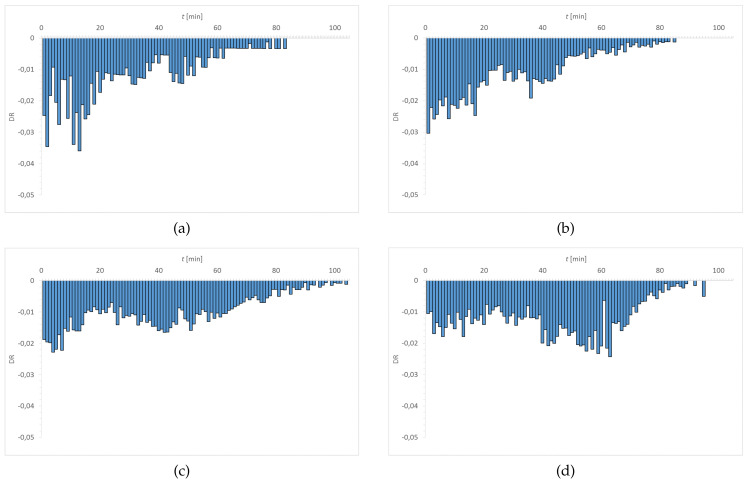
Distribution of DR values as a function of time for Senofilcon A: (**a**) initial state, (**b**) incubated in CLS, (**c**) incubated in CLS & ED, and (**d**) incubated in ED.

Figure 7, Figure 8, Figure 9 and Figure 10 show the distribution of DR values as a function of time. The obtained charts have a characteristic shape. The highest values of DR corresponded to the first 30–40 min. The exception is the graph obtained for Senofilcon A incubated in ED. In this material, the highest dehydration rate was observed in the time interval of 40–60 min.

Water loss was most rapid in Senofilcon A in the initial state (highest DR values in the phase I), whereas the slowest loss of water was in Etafilcon A incubated in CLS & ED (the lowest DR values in the phase I). In the initial state, dehydration was more stable for hydrogel contact lenses (more homogeneous DR profile) than for silicone hydrogel lenses. In addition, in the initial state, Narafilcon A and Senofilcon A yielded similar DR profiles, showing comparable dehydration peaks during the whole tested time. Omafilcon A in initial state presented one characteristic peak at 10–11 min, whereas Etafilcon A showed a double peak. One of them was observed in the phase I and the other at 40 min of the study. There was no observed significant effect of incubation in ophthalmic fluids on DR values (*p* > 0.05) but it was noticed that storage influences dehydration time.

### 3.2. Wettability and Surface Free Energy (SFE)

**Figure 11:**
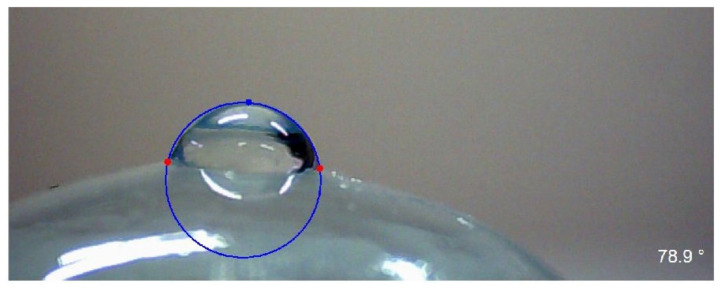
Water contact angle measurement for Narafilcon A.

The measurement results of the water CA are shown in Table 3. Presented results are the arithmetic mean of all measurements made for a given research group. Statistically significant differences in CA values between the hydrogel and silicone hydrogel contact lenses were observed (*p* < 0.001). In the initial state, the values of CA for hydrogel contact lenses were smaller than for silicone hydrogel lenses. For Etafilcon A was it 40.3 ± 6.9${}^{{}^{\circ}}$ and for Omafilcon A 43.7 ± 7.0${}^{{}^{\circ}}$, whereas for Narafilcon A was 72.2 ± 9.7${}^{{}^{\circ}}$ and for Senofilcon A 76.3 ± 8.1${}^{{}^{\circ}}$. An example of the CA measurement is shown in Figure 11.

The surfaces of all tested contact lenses were hydrophilic. The incubation environment notably influences the CA values but the character of a surface did not change (stayed hydrophilic-the value of CA remained less than 90${}^{{}^{\circ}}$). The most significant difference in the value of the water CA was observed for the contact lenses incubated in the ED (*p* < 0.001). In this case, it was not possible to deposit the water drops on the surface because the CA decreased significantly (the surface became more hydrophilic).

When the contact lenses were incubated in the CLS, the CA increased slightly, with a greater difference being seen with the hydrogel lenses. For the contact lenses stored in CLS & ED, an increase in the value of the CA was observed for Omafilcon A, Narafilcon A, and Senofilcon A. The value of the CA for Etafilcon A has not changed in a statistically significant way after this incubation (*p* = 0.33).

The measurement of the CA was used for further analysis to calculate the SFE. Table 4 and Table 5 present a summary of the values of the SFE calculated using the following methods: Owens–Wendt, Wu, Neumann, and Neumann–Kwok. The obtained SFE values, calculated using various methods, were similar for each material; however, in the Wu method, the results were higher than those obtained using the other models.

Differences in the value of SFE between the hydrogel and silicone hydrogel contact lenses were observed. Higher SFE values were found for hydrogel contact lenses, e.g., according to the Owens–Wendt method; they ranged from 54.45 ± 6.56 mJ/m${}^{2}$ to 58.09 ± 4.86 mJ/m${}^{2}$, while the SFE values for the silicone hydrogel contact lenses were from 32.86 ± 3.47 mJ/m${}^{2}$ to 35.33 ± 6.56 mJ/m${}^{2}$. Incubation in all tested environments caused a decrease in the value of SFE but these differences were in most cases statistically insignificant. A general tendency was observed that for silicone hydrogel materials the value of the dispersive component of SFE was greater than the value of the polar component, whereas for hydrogel materials, in most of the analyzed cases, higher values were assumed by the polar component; however, for Omafilcon A this relationship is unclear.

In the case of samples incubated in ED, it was only possible to calculate the dispersive component of the SFE. However, it was not feasible to calculate the polar component and thus the SFE. This was because it was not possible to deposit the water drops on the surface of the material. For the dispersive component, a decrease of the value after incubation in ED for all types of contact lenses was observed.

## 4. Discussion

Four types of contact lenses were tested. All tested contact lenses had the same back vertex power. Because of this, any differences observed in the dehydration process result directly from the type of contact lens material or the influence of the incubation environment. According to Willcox et al. [16], there is inconsistent evidence that ED or CLS can significantly affect or maintain the wettability of contact lenses during wear. Therefore, the study attempts to evaluate the influence of the incubation environment on the surface properties of contact lenses.

This study showed that hydrogel and silicone hydrogel contact lenses yielded dissimilar dehydration profiles. Hydrogel materials presented a more homogenous distribution of DR values than silicone hydrogel contact lenses. The DR curves showed similar dehydration profiles for Narafilcon A and Senofilcon A in the initial state. This could have been due to the analogous chemical composition of these silicone hydrogel materials. Additionally, Senofilcon A in the initial state seems to be the least resistant to the dehydration process (the most rapid changes of DR values). This statement confirms the results of a study by Lira et al. [62] that analyzes the influence of different lens care systems in surface roughness and refractive index (RI) of contact lenses. According Lira’s research, Senofilcon A always increases the refractive index, which may be interpreted as a decrease in water content. Note that the dehydration profile over time of the tested contact lenses showed a different dehydration curve depending on the stored conditions. Martín-Montañez et al. [34] in their in vitro studies found a significant influence of air humidity and the type of contact lens material on the DR value and the interpretation of the DR profile. In our research, uniform air humidity conditions were maintained to eliminate the influence of this factor on the obtained results.

The most significant changes were observed for contact lenses incubated in ED. In all tested cases (except Senofilcon A incubated in ED), the quickest water loss was during the first minutes of the study. A similar feature was already described by González-Méijome et al. [30] and Martín-Montañez et al. [34]. González-Méijome et al. [30] reported that high EWC materials presented a significantly longer phase I (44.50 ± 10.97 min) compared to medium EWC (22.75 ± 7.32 min) and low EWC (12.00 ± 3.91 min). Based on our research, it is not possible to clearly confirm this relationship, and phase I lasted about 40 min in all tested cases. According to Krysztofiak et al. [63], the time needed for Senofilcon A dehydration is approximately 72 min, while this study has shown that it is about 69 min. In other work, Krysztofiak et al. [52] reported that Narafilcon A has a shorter phase I than conventional hydrogels. According to Morgan et al. [64], the EWC value for Etafilcon A is 58.1% ± 0.6%, which is similar to our result 52.7% ± 2.1%. The results obtained by Efron et al. [18] suggest that with Etafilcon A the loss of oxygen performance is proportional to the amount of water lost through the contact lens.

According to Sekine et al. [65], three phases of dehydration reflect water structure in the material, which additionally influences its other properties [66]. Krysztofiak et al. [52] assumed that in phase I bulk water molecules evaporate. As they do not interact with the material, dehydration follows rapidly. This statement cannot be directly concluded from the results obtained in the presented study. Dehydration of loosely bound water occurs in phase II. In phase III, the material is balanced with the environment and dehydration is complete. This is because the tightly bound water molecules produce strong bindings. According to Fornasiero et al. [67], a thin glassy layer may be present on the surface of the contact lens when exposed to air, which limits further evaporation. Accumulated deposits can be found on dehydrated areas of the contact lenses, which in turn may reduce wettability and cause discomfort [68].

The stabilization time (phase III of dehydration) shows when the contact lens has almost lost all the water content. Omafilcon A reached its stabilization time the fastest and Senofilcon A the slowest. The results presented in this work indicated that the stabilization time was longer for contact lenses after incubation in ophthalmic fluids (except Etafilcon A in CLS & ED and Senofilcon A in ED). Marx et al. [28] in their studies found that CLS-containing surfactant wetting agents delay the surface dehydration of silicone hydrogel contact lenses, which is consistent with our study because incubation of the lenses in the ED caused prolongation of the dehydration time.

Alves et al. [69] investigated the interaction between contact lenses and the CLS. In their work, they suggest that the optical variables of CLS and contact lenses themselves changed after storage. For example, after storage in the different CLS, the transmittance of each material (i.a., Senofilcon A, Omafilcon A) exhibited significant changes. It was probably related to the biophysical and biochemical interactions between the ingredients and the release of some polymeric compounds. Alves concluded that HA enhances water retention and does not influence the contact lenses optical properties. This is partly in line with our research because the dehydration process was longer for the contact lenses incubated in the ED containing HA. The conducted research confirmed that the dynamic of dehydration depends both on the chemical composition and the type of water environment but the chemical composition of the contact lenses has a greater influence. The initial EWC value is also important and affects the DR value. Taking into account the relatively long dehydration time, the DR distribution and the lowest value of water CA, Etafilcon A could be considered the most advantageous in clinical use.

Values of CA and SFE were used to indicate differences in surface properties of materials. Large research group (100 measurements of CA for each case) contributed to the statistical significance of the results described. It was observed that the water CA achieved lower values for hydrogel contact lenses. Higher values of this parameter for silicone hydrogel contact lenses may be related to the bulk content of the Si-rich hydrophobic phase reaching over 30% [70]. Similar studies were carried out by Iwashita et al. [71], who made the opposite conclusion that CA values associated with the silicone hydrogel contact lenses were significantly lower than those associated with the hydrogel contact lenses. In addition, storing the contact lenses in HA increased CA values.

Our studies showed an increase of hydrophobicity for contact lenses stored in CLS and CLS & ED (except Etafilcon A in CLS & ED) but surfaces remained hydrophilic nonetheless, whereas hydrophilicity increased for contact lenses incubated in ED because there was no possibility to sessile a water drop. In addition, Lira et al. [72] found that there were interactions between the contact lens material and the CLS, which can significantly influence on the surface wettability of contact lenses.

The obtained values of CA for Senofilcon A were in good agreement with the results obtained by Lira et al. [45]. The differences were observed in the values of the CA for Etafilcon A, but it was within the measurement error. Lira et al. obtained values of CA in the initial state for Etafilcon A 49.6 ± 3.0${}^{{}^{\circ}}$ and for Senofilcon A 78.9 ± 2.5${}^{{}^{\circ}}$. They also found interactions between contact lens material and CLS that may have an important role in the hydrophobicity of the contact lens surface. Bruinsma et al. [73] analyzed Etafilcon A for which the value of CA was 45${}^{{}^{\circ}}$. Tonge’s et al. [74] study showed that the Etafilcon A lenses did not show significant changes in wettability during the initial 4h wear period. Yamasaki et al. [75] investigated the effects of hyaluronic acid and low molecular weight hyaluronic acid on contact lenses. According to these results, HA increases the hydrophilicity of the lens surface. This effect was more durable when using solutions containing low molecular weight hyaluronic acid. Our research also showed an increase in the hydrophilicity of the surface of contact lenses incubated in ED.

Eftimov et al. [15,76] studied the influence of air exposure time on the water CA of silicone hydrogel contact lenses and reported that in the initial state the values of CA for Narafilcon A and Senofilcon A were 66.7${}^{{}^{\circ}}$ and 68.6${}^{{}^{\circ}}$, respectively. It slightly differed from our results (72.2 ± 9.7${}^{{}^{\circ}}$ for Narafilcon A and 76.3 ± 8.1${}^{{}^{\circ}}$ for Senofilcon A), but their results were within our error limit. Menzies [77] reported that in the initial state water CA for Omafilcon A was 93${}^{{}^{\circ}}$, for Narafilcon A was 102${}^{{}^{\circ}}$, and for Etafilcon A was 50${}^{{}^{\circ}}$. The inconsistency of the results with other tests may ensue from many circumstances, such as the use of a different measurement method or the influence of the environment [78].

Studies have shown that a greater water CA value was found in silicone hydrogel contact lenses (more hydrophobic). The main problem with silicone-based contact lenses is the inherent hydrophobicity of the siloxane groups, which greatly inhibits the development of silicone hydrogel contact lenses. The hydrophobicity leads to problems such as decreased surface wettability and increased lipid interaction. However, the main advantage of silicone hydrogel materials over hydrogels is their greater oxygen permeability, which results in a reduction of corneal hypoxia [79]. According to Wolffsohn et al. [80] the lens surface wetting is not only related to lens properties, but also the interaction between an individual’s ocular surface including their tear film, and interaction with the lens. Therefore, further in vivo studies are required for the clinical evaluation of our results.

Studies describing the connection between bacterial adhesion and surface wettability exhibit inconsistent results. Some studies show that bacterial adhesion is higher for hydrophobic substrates, and other works deliver information about increased biofilm growth on highly hydrophilic surfaces [47,81]. Values of water CA in the range from 40${}^{{}^{\circ}}$ to 70${}^{{}^{\circ}}$ are recognized as most opportunely for cell adhesion on polymer materials [78]. Based on this information and our results one would assume that hydrogel contact lenses are more convenient for cell adhesion than silicone hydrogel lenses. However, it cannot be clearly stated on the basis of the presented research. Additional bacterial tests should be performed to verify this statement. Conversely, many studies showed that the hydrophobic character of the surface may increase bacterial adhesion which suggests that silicone hydrogel contact lenses had a higher extent of bacterial adhesion [47,81,82,83].

According to Campbell et al. [84], the SFE of material shows how CA analysis is capable of providing supplementary surface characterization. Therefore, in this study, the SFE was calculated from the CA. Taking into account all four approaches used for calculating the SFE values, it should be noted that despite various assumptions each gave comparable results. The research of Lin et al. [85] reported that the SFE values for Etafilcon A, Omafilcon A, and Senofilcon A were 44.43 mJ/m${}^{2}$, 57.33 mJ/m${}^{2}$, 49.13 mJ/m${}^{2}$, respectively. Only for Omafilcon A are the results presented in our study similar. In the case of Etafilcon A, the obtained value of SFE was higher than given by Lin. However, for Senofilcon A value of this parameter was lower.

## 5. Conclusions

The presented results provide information about the influence of selected ophthalmic fluids on the in vitro wettability, dehydration, and surface properties of contact lenses. Dissimilarity found in the contact lenses’ dehydration profiles can be clarified by differences in the chemical composition of the miscellaneous materials studied. Our work investigated the effect of only one selected contact lens solution and one type of eye drops on the properties of the lenses.

Taking into account the research of other biomaterials and the few works dealing with the calculation of SFE for contact lenses, an attempt was made to adapt this method to the description of contact lenses. These studies were combined with frequently used dehydration studies to make a broad characterization of the material. Calculation of SFE is very useful because it can be used to estimate the possibility of bacterial adhesion on contact lenses surfaces. It is clinically important to maintain the wettability of the contact lenses over a longer period of time to maintain eye health and wearer comfort. Further, in vivo studies would be needed to understand better the clinical impact of ophthalmic fluids on surface properties, wettability, and dehydration of contact lenses. In order to complete the analysis, the presented research should be extended to oxygen permeability, bacterial adhesion, and dehydration at different humidity or airflow conditions.

## Figures and Tables

**Table 1:** Parameters and chemical composition of studied contact lenses [32,49,50].

Material	EWC [%]	Oxygen Permeability [Dk/t]	FDA Group	Chemical Composition
Etafilcon A	58	23.8–28	IV	HEMA, MAA, EGDMA, TMPTMA, PVP
Omafilcon A	60	37	II	HEMA, PC
Narafilcon A	46	118	V	HEMA, mPDMS, DMA, TEGDMA, PVP, siloxane monomer
Senofilcon A	38	103–147	I	mPDMS, DMA, HEMA, siloxane macromere, PVP, TEGDMA

HEMA—poly-2-hydroxyethyl methacrylate; MAA—methacrylic acid; EGDMA—ethylene glycol dimethacrylate; TMPTMA-trimethylolpropane trimethacrylate; PVP—polyvinylpyrrolidone; PC—phosphorylcholine; mPDMS—monofunctional polydimethylsiloxane; DMA—N,N-dimethylacrylamide; TEGDMA—tetraethyleneglycol dimethacrylate.

**Table 2:** SFE and its components for the liquids used in contact angle experiment (mJ/m${}^{2}$) [53].

Measuring Liquid	$\gamma_{L}$	$\gamma_{L}{}^{d}$	$\gamma_{L}{}^{p}$
Distilled water	72.8	21.8	51.0
Diiodomethane	50.8	50.8	0.0

**Table 3:** Values of the water contact angle for tested materials.

Material	Contact Angle (${}^{{}^{\circ}}$)			
	Initial State	After Incubation		
		in CLS	in CLS & ED	in ED
Etafilcon A	40.3 ± 6.9	44.8 ± 5.6	40.6 ± 3.2	-
Omafilcon A	43.7 ± 7.0	46.7 ± 9.3	45.3 ± 6.9	-
Narafilcon A	72.2 ± 9.7	73.8 ± 5.9	75.9 ± 5.3	-
Senofilcon A	76.3 ± 8.1	78.6 ± 5.6	81.6 ± 8.9	-

CLS: contact lens solution; ED: eye drops.

**Table 4:** Comparison of the SFE and its components (mJ/m${}^{2}$) calculated using Owens–Wendt method.

Material	Environment	Owens-Wendt		
		$\gamma_{S}^{d}$	$\gamma_{S}^{p}$	$\gamma_{S}$
Etafilcon A	Initial state	26.35 ± 4.73	31.74 ± 5.42	58.09 ± 4.86
	CLS	25.13 ± 4.09	29.68 ± 4.48	54.81 ± 4.12
	CLS & ED	26.63 ± 3.37	31.35 ± 3.26	57.98 ± 2.22
	ED	21.20 ± 3.11	-	-
Omafilcon A	Initial state	28.03 ± 5.21	28.29 ± 5.36	56.32 ± 4.86
	CLS	26.77 ± 5.28	27.68 ± 7.35	54.45 ± 6.56
	CLS & ED	28.93 ± 4.89	27.02 ± 5.85	55.95 ± 4.79
	ED	22.59 ± 3.89	-	-
Narafilcon A	Initial state	21.37 ± 5.43	13.96 ± 5.85	35.33 ± 6.56
	CLS	22.59 ± 3.57	12.03 ± 3.81	34.62 ± 3.55
	CLS & ED	21.72 ± 3.65	11.14 ± 3.59	32.86 ± 3.47
	ED	19.77 ± 4.42	-	-
Senofilcon A	Initial state	23.55 ± 5.67	10.49 ± 5.08	34.04 ± 5.65
	CLS	25.69 ± 3.70	8.07 ± 2.95	33.75 ± 3.78
	CLS & ED	26.58 ± 4.76	6.81 ± 4.27	33.39 ± 5.13
	ED	21.41 ± 4.26	-	-

CLS: contact lens solution, ED: eye drops.

**Table 5:** Comparison of the SFE and its components (mJ/m${}^{2}$) calculated using Wu, Neumann, and Neumann–Kwok methods.

Material	Environment	Wu			Neumann	Neumann–Kwok
		$\gamma_{S}^{d}$	$\gamma_{S}^{p}$	$\gamma_{S}$	$\gamma_{S}$	$\gamma_{S}$
Etafilcon A	Initial state	28.61 ± 4.03	32.22 ± 4.16	60.83 ± 4.50	59.10 ± 3.74	58.80 ± 3.85
	CLS	27.57 ± 3.48	30.26 ± 3.41	57.83 ± 3.80	56.69 ± 3.15	56.33 ± 3.24
	CLS & ED	28.84 ± 2.87	31.91 ± 2.31	60.75 ± 2.19	59.00 ± 1.73	58.71 ± 1.78
	ED	24.24 ± 2.64	-	-	-	-
Omafilcon A	Initial state	30.05 ± 4.47	29.59 ± 4.07	59.64 ± 4.60	57.11 ± 3.80	56.76 ± 3.91
	CLS	28.98 ± 4.51	28.87 ± 5.79	57.85 ± 5.81	55.78 ± 5.45	55.40 ± 5.60
	CLS & ED	30.82 ± 4.21	28.67 ± 4.47	59.49 ± 4.38	56.49 ± 4.08	56.12 ± 4.19
	ED	25.41 ± 3.30	-	-	-	-
Narafilcon A	Initial state	24.37 ± 4.62	16.57 ± 4.99	40.94 ± 6.03	40.32 ± 5.98	39.72 ± 5.94
	CLS	25.41 ± 3.04	15.13 ± 3.24	40.54 ± 3.29	39.37 ± 3.66	38.74 ± 3.62
	CLS & ED	24.67 ± 3.11	14.28 ± 3.00	38.95 ± 3.20	38.01 ± 3.31	37.38 ± 3.28
	ED	23.02 ± 3.76	-	-	-	-
Senofilcon A	Initial state	26.22 ± 4.82	13.78 ± 4.32	40.00 ± 5.27	37.80 ± 5.01	37.20 ± 4.97
	CLS	28.04 ± 3.14	11.86 ± 2.73	39.90 ± 3.61	36.33 ± 3.50	35.73 ± 3.45
	CLS & ED	28.81 ± 4.06	10.44 ± 4.22	39.25 ± 5.01	34.47 ± 5.55	33.92 ± 5.44
	ED	24.41 ± 3.61	-	-	-	-

CLS: contact lens solution, ED: eye drops.

## Data Availability

Date sharing is not applicable.

## References

[1] Vidal-Rohr M., Wolffsohn J.S., Davies L.N., Cerviño A. (2018). Effect of contact lens surface properties on comfort, tear stability and ocular physiology. Contact Lens Anterior Eye.

[2] Tranoudis I., Efron N. (2004). In-eye performance of soft contact lenses made from different materials. Contact Lens Anterior Eye.

[3] Roba M., Duncan E.G., Hill G.A., Spencer N.D., Tosatti S.G.P. (2011). Friction Measurements on Contact Lenses in Their Operating Environment. Tribol. Lett..

[4] Guillon M., Patel T., Patel K., Gupta R., Maissa C.A. (2019). Quantification of contact lens wettability after prolonged visual device use under low humidity conditions. Contact Lens Anterior Eye.

[5] Brygoła R., Sęk S., Sokołowski M., Kowalczyk-Hernández M., Pniewski J. (2018). Limits in measurements of contact lens surface profile using atomic force microscopy. Colloids Surf. B Biointerfaces.

[6] Samsom M., Iwabuchi Y., Sheardown H., Schmidt T.A. (2018). Proteoglycan 4 and hyaluronan as boundary lubricants for model contact lens hydrogels. J. Biomed. Mater. Res. B Appl. Biomater..

[7] Yu Y., Macoon R., Chauhan A. (2019). Improving wettability and lubricity of commercial contact lenses by polymerizing a thin film of dimethylacryamide. Colloids Surf. A Physicochem. Eng. Asp..

[8] Jones L., May C., Nazar L., Simpson T. (2002). In Vitro evaluation of the dehydration characteristics of silicone hydrogel and conventional hydrogel contact lens materials. Contact Lens Anterior Eye.

[9] Rajchel D., Krysztofiak K., Szyczewski A. (2016). Influence of sodium hyaluronate on dehydration and water distribution in soft contact lenses. Opt. Appl..

[10] Filipecki J., Sitarz M., Kocela A., Kotynia K., Jeleń P., Filipecka K., Gawęda M. (2014). Studying functional properties of hydrogel and silicone–hydrogel contact lenses with PALS, MIR and Raman spectroscopy. Spectrochim. Acta A Mol. Biomol. Spectrosc..

[11] Levitt A.E., Galor A., Chowdhury A.R., Felix E.R., Sarantopoulos C.D., Zhuang G.Y., Patin D., Maixner W., Smith S.B., Martin E.R. (2017). Evidence that Dry Eye Represents a Chronic Overlapping Pain Condition. Mol. Pain.

[12] McMonnies C.W. (2021). Could contact lens dryness discomfort symptoms sometimes have a neuropathic basis?. Eye Vis..

[13] Situ P., Simpson T.L., Jones L.W., Fonn D. (2010). Effects of Silicone Hydrogel Contact Lens Wear on Ocular Surface Sensitivity to Tactile, Pneumatic Mechanical, and Chemical Stimulation. Investig. Ophthalmol. Vis. Sci..

[14] Murphy P.J., Patel S., Marshall J. (2001). The Effect of Long-term, Daily Contact Lens Wear on Corneal Sensitivity. Cornea.

[15] Eftimov P., Yokoi N., Peev N., Georgiev G.A. (2019). Impact of Air Exposure Time on the Water Contact Angles of Daily Disposable Silicone Hydrogels. Int. J. Mol. Sci..

[16] Willcox M., Keir N., Maseedupally V., Masoudi S., McDermott A., Mobeen R., Purslow C., Santodomingo-Rubido J., Tavazzi S., Zeri F. (2021). BCLA CLEAR—Contact lens wettability, cleaning, disinfection and interactions with tear. Contact Lens Anterior Eye.

[17] Lin C., Cho H., Yeh Y., Yang M. (2015). Improvement of the surface wettability of silicone hydrogel contact lenses via layer-by-layer self-assembly technique. Colloids Surf. B Biointerfaces.

[18] Efron N., Morgan P.B. (1999). Hydrogel contact lens dehydration and oxygen transmissibility. Clao J..

[19] Pritchard N., Fonn D. (1995). Dehydration, lens movement and dryness ratings of hydrogel contact lenses. Ophthalmic Physiol. Opt..

[20] Andrasko G., Schoessler J. (1980). The effect of humidity on the dehydration of soft contact lenses on the eye. Int. Contact Lens Clin..

[21] Brennan N., Efron N., Bruce A., Duldig D., Russo N. (1988). Dehydration of hydrogel lenses: Environmental influences during normal wear. Am. J. Optom. Physiol. Opt..

[22] Efron N., Brennan N. (1987). The clinical relevance of hydrogel lens water content. Contact Lens Anterior Eye.

[23] Caló E., Khutoryanskiy V.V. (2015). Biomedical applications of hydrogels: A review of patents and commercial products. Eur. Polym. J..

[24] Brennan N., Efron N. (1987). Hydrogel lens dehydration: A material dependent phenomenon?. Contact Lens Forum.

[25] Larsen D., Huff J., Holden B. (1990). Proton NMR relaxation in hydrogel contact lenses: Correlation with in vivo lens dehydration data. Curr. Eye Res..

[26] Benz P., Ors J. (1997). New materials demand more accurate measurements of performance. Spectrum.

[27] Andrasko G. (1983). Hydrogel dehydration in various environments. Int. Contact Lens Clin..

[28] Marx S., Sickenberger W. (2017). A novel In-Vitro method for assessing contact lens surface dewetting: Noninvasive keratograph dry-up time (NIK-DUT). Contact Lens Anterior Eye.

[29] Wechsler S., Johnson M., Businger U. (1983). In Vivo hydration of hydrogel lenses—The first hour. Int. Contact Lens Clin..

[30] González-Méijome J.M., López-Alemany A., Almeida J.B., Parafita M.A., Refojo M.F. (2007). Qualitative and quantitative characterization of the in vitro dehydration process of hydrogel contact lenses. Biomed. Mater. Res. B Appl. Biomater..

[31] Insua Pereira E., Lira M. (2018). Comfort, Ocular Dryness, and Equilibrium Water Content Changes of Daily Disposable Contact Lenses. Eye Contact Lens.

[32] Krysztofiak K. (2015). Badanie procesu dehydratacji w hydrożelowych i silikonowo-hydrożelowych soczewkach kontaktowych. Ph.D. Thesis.

[33] Kyritsis A., Pissis P., Gómez Ribelles J.L., Monleón Pradas M. (1995). Polymer-water interactions in poly(hydroxyethyl acrylate) hydrogels studied by dielectric, calorimetric and sorption isotherm measurements. Polym. Gels Netw..

[34] Martín-Montañez V., López-Miguel A., Arroyo C., Mateo M.E., González-Méijome J.M., Calonge M., González-García M.J. (2014). Influence of environmental factors in the in vitro dehydration of hydrogel and silicone hydrogel contact lenses. J. Biomed. Mater. Res. B Appl. Biomater..

[35] Calonge M. (2001). The treatment of dry eye. Surv. Ophthalmol..

[36] Stahl U., Willcox M., Stapleton F. (2010). Role of hypo-osmotic saline drops in ocular comfort during contact lens wear. Contact Lens Anterior Eye.

[37] Lapčík L., De Smedt S., Demeester J., Chabreček P. (1998). Hyaluronan: Preparation, structure, properties, and applications. Chem. Rev..

[38] Nashida T., Nakamura M., Mishima H., Otori T. (1991). Hyaluronan stimulates corneal epithelial migration. Exp. Eye Res..

[39] Rah M.J. (2011). A review of hyaluronan and its ophthalmic applications. Optometry.

[40] Aragona P., Di Stefano G., Ferreri F., Spinella R., Stilo A. (2002). Sodium hyaluronate eye drops of different osmolarity for the treatment of dry eye in Sjögren’s syndrome patients. Br. J. Ophthalmol..

[41] Brjesky V.V., Maychuk Y.F., Petrayevsky A.V., Nagorsky P.G. (2014). Use of preservative-free hyaluronic acid (Hylabak) for a range of patients with dry eye syndrome: Experience in Russia. Clin. Ophthalmol..

[42] Cazzaniga G., Ottobelli M., Ionescu A., Garcia-Godoy F., Brambilla E. (2015). Surface properties of resin-based composite materials and biofilm formation: A review of the current literature. Am. J. Dent..

[43] Lam C.N.C., Kim N., Hui D., Kwok D.Y., Hair M.L., Neumann A.W. (2001). The effect of liquid properties to contact angle hysteresis. Colloids Surf. A Physicochem. Eng. Asp..

[44] Simpson J.T., Hunter S.R., Aytug T. (2015). Superhydrophobic materials and coatings: A review. Rep. Prog. Phys..

[45] Lira M., Silva R. (2018). Contact lens care solutions and their influence on contact lens hydrophobicity. Contact Lens Anterior Eye.

[46] Figura P. (2015). Powierzchnia soczewek kontaktowych a bezpieczeństwo i komfort użytkownika. OphthaTherapy.

[47] Giraldez M.J., Resua C.G., Lira M., Oliveira M.E., Magariños B., Toranzo A.E., Yebra-Pimentel E. (2010). Contact lens hydrophobicity and roughness effects on bacterial adhesion. Optom. Vis. Sci..

[48] Yu Y., Hsu K.H., Gharami S., Butler J.E., Hazra S., Chauhan A. (2020). Interfacial polymerization of a thin film on contact lenses for improving lubricity. J. Colloid Interface Sci..

[49] Galas S., Copper L. (2016). Oxygen permeability of the pigmented material used in cosmetic daily disposable contact lenses. Clin. Ophthalmol..

[50] Lewandowska M., Jasińska-Kwaśnik W., Jóźwik A., Siedlecki D. (2015). Measurement of oxygen permeability of contact lenses based on analysis of porosity. Int. J. Eng. Sci. Technol..

[51] Burgos-Fernández F.J., Guaus E., Martínez C., Vilaseca M. (2019). Terahertz-based system for dehydration analysis of hydrogel contact lenses. Opt. Appl..

[52] Krysztofiak K., Szyczewski A. (2014). Study of dehydration and water states in new and worn soft contact lens materials. Opt. Appl..

[53] Żenkiewicz M. (2007). Methods for the calculation of surface free energy of solids. J. Achiev. Mater. Manuf..

[54] Owens D.K., Wendt R.C. (1969). Estimation of the Surface Free Energy of Polymers. J. Appl. Polym. Sci..

[55] Liber-Kneć A., Łagan S. (2021). Surface Testing of Dental Biomaterials—Determination of Contact Angle and Surface Free Energy. Materials.

[56] Lotfi M., Nejib M., Naceur M., Pignatello R. (2013). Cell adhesion to biomaterials: Concept of biocompatibility. Advances in Biomaterials Science and Biomedical Applications.

[57] Wu S. (1971). Calculation of Interfacial Tension in Polymer Systems. J. Polym. Sci. Part C.

[58] Hejda F., Solař P., Kousal J. (2010). Surface Free Energy Determination by Contact Angle Measurements–A Comparison of Various Approaches. Proceedings of the 19th Annual Conference of Doctoral Students—WDS’10; Proceedings of Contributed Papers, Part III.

[59] Neumann A.W., Good R.J., Hope C.J., Sejpol M. (1974). An equation-of-state approach to determine surface tensions of low-energy solids from contact angles. J. Colloid Interface Sci..

[60] Li D., Neumann A.W. (1990). A reformulation of the equation of state for interfacial tensions. J. Colloid Interface Sci..

[61] Kwok D.Y., Neumann A.W. (1999). Contact angle measurement and contact angle interpretation. Adv. Colloid Interface Sci..

[62] Lira M., Franco S., Vazquez-Dorrio J.B., Real Oliveira M.E., Costa M.F. (2014). Surface roughness and refractive index changes in contact lens induced by lens care systems. Eye Contact Lens.

[63] Krysztofiak K., Płucisz M., Szyczewski A. (2012). The influence of wearing on water states and dehydration of silicone-hydrogel contact lenses. Eng. Biomater..

[64] Morgan P.B., Efron N. (2003). In Vivo Dehydration of Silicone Hydrogel Contact Lenses. Eye Contact Lens.

[65] Sekine Y., Ikeda-Fukazawa T. (2009). Structural changes of water in a hydrogel during dehydration. J. Chem. Phys..

[66] Tranoudis I., Efron N. (2004). Water properties of soft contact lens materials. Contact Lens Anterior Eye.

[67] Fornasiero F., Krull F., Radke C.J., Prausnitz J.M. (2005). Diffusivity of water through a HEMA-based soft contact lens. Fluid Phase Equilibria.

[68] French K. (2005). Contact lens material properties part 1—Wettability. Optician.

[69] Alves M., Castanheira E.M.S., Lira M. (2021). Interactions between contact lenses and lens care solutions: Influence in optical properties. Contact Lens Anterior Eye.

[70] Curtin S., Seitz M., Ouyang M., Tomic K., Wiseman M., Vanwersch H. (2013). Characterization of contact lenses through oxygen permeability, equilibrium water content, and silicone content. Investig. Ophthalmol. Vis. Sci..

[71] Iwashita H., Itokawa T., Suzuki T., Okajima Y., Kakisu K., Hori Y. (2021). Evaluation of In Vitro Wettability of Soft Contact Lenses Using Tear Supplements. Eye Contact Lens.

[72] Lira M., Silva R. (2017). Effect of Lens Care Systems on Silicone Hydrogel Contact Lens Hydrophobicity. Eye Contact Lens.

[73] Bruinsma G.M., Rustema-Abbing M., de Vries J., Stegenga B., van der Mei H.C., van der Linden M.L., Hooymans J.M., Busscher H.J. (2002). Influence of wear and overwear on surface properties of etafilcon A contact lenses and adhesion of Pseudomonas aeruginosa. Investig. Ophthalmol. Vis. Sci..

[74] Tonge S., Jones L., Goodall S., Tighe B. (2001). The ex vivo wettability of soft contact lenses. Curr. Eye Res..

[75] Yamasaki K., Drolle E., Nakagawa H., Hisamura R., Ngo W., Jones L. (2021). Impact of a low molecular weight hyaluronic acid derivative on contact lens wettability. Contact Lens Anterior Eye.

[76] Eftimov P.B., Yokoi N., Peev N., Paunski Y., Georgiev G.A. (2021). Relationships between the material properties of silicone hydrogels: Desiccation, wettability and lubricity. J. Biomater. Appl..

[77] Menzies K.L. (2010). In Vitro Analysis of Wettability and Physical Properties of Blister Pack Solutions of Hydrogel Contact Lenses. Ph.D. Thesis.

[78] Xu L., Siedlecki C.A. (2007). Effects of surface wettability and contact time on protein adhesion to biomaterial surfaces. Biomaterials.

[79] Fagehi R., Pearce E.I., Oliver K., Abusharha A.A., Tomlinson A. (2017). Care solution effects on contact lens in vivo wettability. Clin. Exp. Optom..

[80] Wolffsohn J., Hall L., Mroczkowska S., Hunt O.A., Bilkhu P., Drew T., Sheppard A. (2015). The influence of end of day silicone hydrogel daily disposable contact lens fit on ocular comfort, physiology and lens wettability. Contact Lens Anterior Eye.

[81] Menzies K.L., Jones L. (2010). The impact of contact angle on the biocompatibility of biomaterials. Optom. Vis. Sci..

[82] Beattie T.K., Tomlinson A., McFadyen A.K., Seal D.V., Grimason A.M. (2003). Enhanced attachment of acanthamoeba to extended-wear silicone hydrogel contact lenses: A new risk factor for infection?. Ophthalmology.

[83] Henriques M., Sousa C., Lira M., Elisabete M., Oliveira R., Oliveira R., Azeredo J. (2005). Adhesion of Pseudomonas aeruginosa and Staphylococcus epidermidis to silicone-hydrogel contact lenses. Optom. Vis. Sci..

[84] Campbell D., Carnell S.M., Eden R.J. (2013). Applicability of Contact Angle Techniques Used in the Analysis of Contact Lenses, Part 1: Comparative methodologies. Eye Contact Lens.

[85] Lin M.C., Svitova T.F. (2010). Contact lenses wettability in vitro: Effect of surface-active ingredients. Optom. Vis. Sci..

